# Does the use of ingredients added to tobacco increase cigarette addictiveness?: A detailed analysis

**DOI:** 10.3109/08958378.2012.663006

**Published:** 2012-03-20

**Authors:** Edward Sanders, Rolf Weitkunat, Aneli Utan, Ruth Dempsey

**Affiliations:** 1 Edward Sanders Scientific Consulting, Neuchâtel, Switzerland; 2 Philip Morris Products SA, Philip Morris International Research & Development, Neuchâtel, Switzerland; 3 Philip Morris International Management SA, Operations Technical Services, Neuchâtel, Switzerland

**Keywords:** Cigarettes, ingredients, addictiveness, nicotine replacement therapy (NRT), meta-analysis

## Abstract

The possibility that ingredients added to tobacco contribute to the addictiveness of cigarette smoking was evaluated by comparing cessation rates of smokers of traditional blended cigarettes to those of smokers of flue-cured cigarettes. Such a comparison is a valid means of assessing cigarette ingredients as traditional blended cigarettes contain ingredients (>20), whereas flue-cured cigarettes contain no or very few ingredients. Separate analysis of 108 treatment groups and 108 control groups from randomized clinical trials of nicotine replacement therapy (NRT) were performed by multiple logistic regressions. The results of these analyses demonstrated slightly higher quit rates for smokers of blended cigarettes (OR = 1.90, 95% Cl 1.70–2.13 and OR = 1.32, 95% Cl 1.14–1.53 for treatment and control groups, respectively).The control groups were also investigated using classification tree analysis from which no difference in quit rates were observed for smokers of either type of cigarette. Further analyses showed that studies that utilized a high level of psychological support in conjunction with NRT produced at least a two-fold increase in quit rates compared to studies that utilized a low level of psychological support. It was also demonstrated that there is a large difference when results were reported by sustained abstinence compared to point prevalence. Additional meta-analyses found the pooled OR for NRT treatment to be in exact agreement with a recent review that assessed the effectiveness of NRT. Overall these results strongly suggest that ingredients used in the manufacture of traditional blended cigarettes do not increase the inherent addictiveness of cigarettes.

## Introduction

The causal relationship between cigarette smoking and a large number of cancers as well as other diseases, particularly cardiovascular disease and chronic obstructive pulmonary disease (COPD), is well established, and there are no rational grounds on which this relationship can be debated (U.S. Department of Health and Human Services, 2010). A key aspect of this harm is the addictive nature of cigarette smoking, generally thought to be primarily caused by the nicotine delivered to the smoke. Nicotine addiction is also discussed in detail in the 2010 US Surgeon General's Report referenced above. Philip Morris International (PMI) agrees that smoking is addictive and that it can be extremely difficult to stop. An important question that has been raised concerns the alleged possibility that tobacco companies knowingly or unknowingly add flavors or other ingredients to their cigarettes that increase the addictive properties of cigarette smoke (Henningfield et al., 2004). The European Scientific Committee on Emerging and Newly Identified Health Risks (SCENIHR) has recently evaluated evidence on the role of tobacco additives on the addictiveness and attractiveness of tobacco products (SCENIHR, 2010). Regarding addictiveness, the Committee concluded that no tobacco additives which are addictive by themselves have so far been identified. However, the Committee recommended additional research, among others, to investigate, in comparable user groups, differences between smokers of brands containing additives (in the continental EU) and smokers of brands using no additives (in the United Kingdom). A possible approach would be to compare cessation rates for smokers of cigarettes with ingredients to smokers of those without ingredients, assuming that the ease or difficulty of quitting smoking can be taken as a measure of cigarette addictiveness. There are currently numerous definitions and measures of tobacco addiction (see, e.g., US Department of Health and Human Services. 2010, p. 109–110). It is clear, however, that one measure of cigarette addictiveness is the degree of difficulty in smoking cessation (SCENIHR, 2010, p. 82; US Department of Health and Human Services, 2010 p. 105–106; Stapleton et al, 1995). Therefore, if there exist cigarette types that differ significantly in the level of ingredients utilized, a comparison of cessation rates in an appropriate setting can provide a strong indication of the possible role of ingredients with respect to cigarette addictiveness.

Indeed there are two major types of cigarettes world-wide: flue-cured cigarettes, which use very few ingredients, and traditional blended cigarettes, which use a number of ingredients. Furthermore, different cigarette markets tend to be heavily dominated by one or the other of these two cigarette types (e.g., the United Kingdom consumes almost exclusively flue-cured cigarettes, whereas the US almost exclusively consumes blended ones). Traditional blended cigarettes utilize three different types of tobacco – flue-cured, burley, and oriental – that are blended together during the manufacturing process, whereas flue-cured cigarettes contain only flue-cured tobacco. Flue-cured tobacco is cured over a generally short period of time at elevated temperatures, while burley and oriental tobaccos are cured at ambient temperatures during a period of about 6 weeks. A major consequence of the difference in curing practices for these two types of tobaccos is that the elevated temperatures used in flue-curing rapidly denature the enzymes in tobacco responsible for sugar metabolism leaving the tobacco with high sugar levels, whereas these sugars are lost during the curing of burley tobaccos. In addition to differences in curing regimens, burley and oriental tobaccos are genetically different both from each other and from flue-cured tobacco. These distinctions are responsible for somewhat different profiles in both the chemical constituents of the tobacco and the smoke constituents when the tobaccos are burned (Davis and Nielsen, 2007).

As indicated above, the difference between flue-cured and traditional blended cigarettes, crucial to the subject of this paper, is the fact that flue-cured cigarettes generally contain no flavoring ingredients, although a small number of substances may be added as humectants and processing aids, whereas traditionalblended cigarettes do use a number of different types of ingredients. One country which consumes almost exclusively flue-cured cigarettes is the United Kingdom. A representative of British American Tobacco testified to the Select Committee on Health of the UK Houses of Parliament that only six ingredients were used in most of the cigarettes they manufactured for sale in the United Kingdom (Select Committee on Health of the UK Houses of Parliament, 2000). Further confirmation of the fact that limited ingredients are used in flue-cured cigarettes, at least in the United Kingdom, can be found in a recent EU Report, which states that 42% of cigarettes sold in the United Kingdom contain no additives, while 48% of cigarettes contain 10 or fewer additives (SCENIHR, 2010, p. 76). Moreover, nine of the top ten brands sold in the United Kingdom in 2010 (ASH, 2011) are flue-cured cigarettes. These nine brands do not contain any casing and flavoring ingredients, according to the information posted on the manufacturers' websites. (Casings are ingredients added during the leaf processing to improve the basic tobacco taste, processing ability and moisture-holding capacity (European Commission Health & Consumer Protection Directorate, 2007)).

Manufactured traditional blended cigarettes contain a number of different types of ingredients, including a mixture of flavors, although each flavor makes up a very small percentage of the total cigarette by weight. PMI lists all ingredients added to cigarettes sold in 92 different countries by both country and specific product (http://www.pmintl-technical-product-information.com/pages/eng/default/aspx). Other manufacturers publish similar lists for their own brands on their own websites. Country-specific data provide maximum use levels, whereas specific product data include the actual level of major ingredients. For example, major ingredients in the German *Marlboro* Red Box include humectants, such as glycerol (1.7%) and propylene glycol (1.3%); casings, such as sugars (sucrose and/or invert sugar, 3.2%), cocoa and cocoa products (0.085%), licorice extract (0.4%), and carob bean and/or extract (0.071%), and binders, such as guar gum (0.3%). In addition, the total amount of natural and artificial flavors used in the German *Marlboro* consists of only 0.004% by weight, and this includes approximately 20 different substances. The one flavor for which the exact level is specified is menthol. Full-flavored *Marlboro* menthol in Germany contains 0.4% menthol by weight. Although blended cigarettes differ in terms of the exact recipe of ingredients used, both among different countries and among different brands within the same country, the quantitative differences are minor, at least with respect to cigarettes manufactured by the large multinational cigarette companies, which comprises the great majority of cigarettes marketed in the countries considered in this paper.

The difference in the use of ingredients by these two types of cigarettes was recently used by Lee et al. (2009) to compare mortality rates from lung cancer and COPD in four countries in which traditional blended cigarettes are essentially exclusively smoked (Austria, Denmark, Germany, and the United States) and three countries in which flue-cured cigarettes are almost exclusively smoked (Australia, Canada, and the United Kingdom). The conclusion of this paper was that, “differences between countries in the rates of two major diseases for which smoking is clearly the predominant cause cannot materially be explained by whether the cigarettes usually smoked in the countries (now and in the past) are flue-cured or blended.” At the time some preliminary analyses were performed to look at cessation rates comparing flue-cured and traditional blended countries. No statistically significant differences were found between these two groups based on average population-based cessation rates. It was also observed, however, that there was a broad range of cessation rates within each group, and it was clear that the group comparisons could not have been particularly robust due to this large variation.

An improved approach would be to use data derived from randomized clinical trials evaluating some type of smoking cessation intervention. Not only, in principle, are all of the people who enroll in such a study committed to stop smoking to at least some degree, but the extent to which individuals actually manage to stop smoking is well documented. Stead etal. (2008) recently published a meta-analysis of 132 clinical trials involving the use of nicotine replacement therapy (NRT) with respect to its effectiveness on smoking cessation. Of these 132 studies, approximately 110 were published in countries where blended cigarettes are smoked, while 20 originated from countries where flue-cured cigarettes are smoked. As a consequence, a comparison of quit rates derived from such studies should be able to determine if smokers of cigarettes with added ingredients are less likely to quit smoking compared to smokers of cigarettes that contain only very limited added ingredients and no added flavors whatsoever; therefore, the setting of a randomized clinical trial would allow the assessment of the impact of the use of ingredients on a smoker's ability to quit and by implication the addictiveness of the type of cigarette smoked.

## Methods

### Selection of data sets

The goal of this analysis was to compare cessation rates for smokers of cigarettes containing added ingredients, including flavors (blended) to those containing few or no ingredients and no flavors (flue-cured), who participated in randomized clinical trials evaluating the effectiveness of NRT intervention. Therefore, initially all such randomized clinical trials should serve as the basis of the data sets to be included. It was assumed that all studies published prior to 2007 were identified in the Stead et al. review, and these studies were supplemented by a literature search focused on identifying additional trials that had been published subsequent to the Stead et al. review (2008). Data sets contained in the review were excluded from further analysis if they had any of the following characteristics, which were likely to bias the results:

1. Data sets in which the subjects did not volunteer. The reason for that was to ensure that only individuals who demonstrated a commitment to quit smoking were included. Therefore, data sets, for example, in which individuals were enrolled by their physician were excluded.
2. Data sets in which mentally ill subjects were preselected given that their ability to stop smoking may be significantly different from individuals without mental impairment.
3. Data sets in which no biochemical validation of smoking cessation was conducted. It has been known for a considerable period of time that self-reported quit status is often exaggerated. For example, Stookey et al. (1987) reported that of the 102 self-reported proclaimed quitters participating in a clinical trial to evaluate smoking cessation methods, validation by exhaled CO_2_ confirmed only 74% of the self-reported quitting status, whereas salivary cotinine confirmed only 55%. The misrepresentation of smoking cessation is also confirmed by a number of studies that were included in the Stead et al. data set, such as Hilberink et al. (2010) and Hays et al. (1999). As a consequence, combining studies that used both types of data could introduce a considerable bias.
4. Data sets that were conducted outside of Europe, the United States, or the four flue-cured countries (Australia, Canada, New Zealand, and the United Kingdom).
5. Data sets that used any type of pharmaceutical intervention, such as bupropion or varenicline. The effectiveness of this type of intervention would appear to be significantly better than any form of NRT; therefore, inclusion of these studies could bias the results.
6. Data sets that did not compare a NRT intervention group with an untreated control group or data sets in which there were differences between the intervention group and the control group besides NRT. Since one of the types of analysis used in this report was meta-analysis on NRT effectiveness, studies without an appropriate control group can not be used.
7. Multicenter data sets where the subjects were chosen from different countries some of which were blended countries and some of which were flue-cured countries.
8. Data sets that did not report the level of smoking cessation for a period of at least 24 weeks. The rationale was that, since smoking cessation rates in such studies will generally decrease over time, including studies that determined these rates at only very short time periods would clearly bias the results. For similar reasons, results available for periods of longer than 16 months were not used.
9. Data sets that did not report the data for the total sample, such as data sets that stratified subjects on the basis of, for example, smoking intensity, but reported results for only heavy smokers.
10. Any data set that did not define the type of abstinence, namely, sustained abstinence or point prevalence.
11. Data sets for which cessation was not the endpoint.

In order to identify further data sets published after the Stead et al. review, a thorough search of both the Cochrane data base and MEDLINE was made using the keywords “nicotine replacement therapy,” “NRT,” and “smoking cessation” from 2006 through July 2010, resulting in a total of 54 potentially relevant articles.

### Data extraction

Data were extracted from the publications meeting the inclusion and exclusion criteria independently by two researchers. Any observed differences were then resolved. In addition to first author, publication year and country where the study was conducted, size of NRT and control groups, and number of successful cessations per group were recorded. The study-specific definition of cessation (sustained versus point prevalence), and the period after which abstinence was determined (with all periods assigned to the dichotomous variable study duration of either 6 or 12 months, and a preference for the latter for data sets reporting data for both periods) was recorded, as well as type of NRT (gum, patch, lozenge, inhaler, and spray) and level of psychological support. The support level “high” was assigned to data sets where more than just reading materials on smoking cessation were distributed to study participants, in which case the recorded support level was “low.” Study quality ratings, as provided by Stead et al., were also recorded and, where data sets were not considered in the Stead et al. review, assigned according to the same criteria (based on effectiveness of randomization procedures as well as their description in the publication). Three categories were adopted, with category A being designated when data sets reported allocation procedures in sufficient detail to ensure that treatment status could not be known or predicted until a participant was enrolled and assigned to a condition; category B being designated when data sets either did not report how randomization was performed or reported it in insufficient detail to ensure that no selection bias had occurred; and category C being designated for data sets that clearly used inferior randomization procedures, such as using the day of the week in which subjects were enrolled.

### Data analysis

Meta-analysis of cessation rates was performed based on study-level NRT effect and precision estimates using MetaAnalyst (version Beta 3.13, Wallace et al., 2009). Since the regression analysis of effects of ingredients on cessation rates estimated odds ratios (OR) (see below), the meta-analysis of NRT effects on cessation rates was also based on OR in order to maintain consistency. Fixed effects estimates were obtained using inverse-variance weighted aggregation, whereas random effects estimates were calculated using the DerSimonian-Laird formulas (Petitti, 2000). In addition to the overall assessment of intervention (NRT) effects, stratified analyses were performed regarding type of NRT, cigarette type, definition of cessation, level of psychological support, study decade, study size (≥300 versus <300 study participants), study quality, and combinations of cigarette type and definition of cessation. It should be noted that in all the analyses conducted that exact date of the study completion could not be determined, since more than half of the publications (56%) provided no relevant information. Of those that did provide information, it was usually the date at the end of the recruitment or of subject enrolment. However, given that there were only very few examples of more than about 1 year between the estimated completion date of the study and its publication, publication date can serve as a surrogate for the actual year of the study. Heterogeneity was assessed through the I^2^ statistic, and pairwise comparisons of log-transformed effects estimates were assessed using Z-tests based on a testwise alpha level of 5%.

Analyses of effects of ingredients (i.e., cigarette type) were conducted by means of logistic regression of cessation rates using SAS (version 9.1). To identify relevant main effects, the following potential predictor variables were considered in forward selection and backward elimination stepwise multiple logistic regression analyses with a testwise alpha level of 5%: cigarette type (blended versus flue-cured as reference, the latter based on findings from Australia, Canada, New Zealand, and the United Kingdom), study size (≥150 versus <150 subjects), study duration (6 versus 12 months), level of psychological support (high versus low), type of abstinence (sustained versus point prevalence), study decade (with the three periods of 1980–1989, 1990–1999, and 2000–2010), and study quality (A, B, or C, as described above). Based on the identified main effects model, for which adjusted OR were calculated in both control and treatment groups data sets separately, screening for interactions was undertaken in control group data. The interaction screening was restricted to the control group to eliminate the potential effect of NRT, which should in principle lead to a simpler interpretation of those interactions that are found. Stepwise multiple logistic regressions (both forward and backward) with all main effects variables and all bivariate interaction terms were undertaken. According to the interaction structure of the final interaction model, effects estimates and confidence intervals based on the covariance matrix were calculated for both predictors participating in a particular interaction, each at the reference level of the other, as well as for the non-reference level(s) of the predictor of primary interest at the non-reference level(s) of the other predictor involved in the interaction. For control and treatment group data combined, conditional multiple logistic regression analysis based on the identified main effects model and additionally including an indicator variable for group (with control group as reference) was performed, taking into account the clustering of pairs of cessation rates within studies.

Classification tree analysis (CTA) (Biggs et al., 1991) in conjunction with main effect and interaction plots were used to identify high-order interactions and to display them in easily interpretable diagrams. Chi-square 5% alpha level tests were used to split the control data set.

## Results

### Studies used in the analysis

Application of the stated exclusion criteria was carried out on both those data sets considered by Stead et al., and the 54 papers identified through searching the literature following the publication of this review. Of the 130 publications (133 data sets) identified as being relevant by Stead et al., 53 (54 data sets) were eliminated as a consequence of the above exclusion criteria. Specifically, one publication was eliminated as a consequence of criterion 1; 1 as a consequence of criterion 2; 19 as a consequence of criterion 3; 7 as a consequence of criterion 4; 3 as a consequence of criterion 5; 18 as a consequence of criterion 6; 3 as a consequence of criterion 7; and 1 as a consequence of criterion 8. It should be noted that data sets from 16 of these publications were not included by Stead et al., in their main analysis that assessed the effect of NRT treatment on smoking cessation. In addition, the publications by Dautzenberg (2001) and Kralikova et al. (2002) were not used, because the results were drawn from abstracts and, in both cases, the results were later published in their entirety. Therefore, of the 133 data sets utilized by Stead et al., a total of 56 were excluded leaving 76 data sets. A number of these data sets described two independent trials. These were not treated independently by Stead et al., but were treated independently in this analysis. As a consequence, the total number of data sets utilized that were drawn from Stead et al. was 91. Of the 54 possibly relevant studies identified by searching the recent literature, 43 were rejected based on the defined exclusion criteria as follows: 3 publications were rejected as a consequence of criterion 2; 10 as a consequence of criterion 3; 4 as a consequence of criterion 4; 2 as a consequence of criterion 5; 11 as a consequence of criterion 6; 1 as a consequence of criterion 7; 5 as a consequence of criterion 8; 1 as a consequence of criterion 9; and 4 as a consequence of criterion 11. In addition, two of these papers were follow-up publications to data sets that had been previously included. Of these additional data sets, three of them, studies by Okuyemi et al. (2007), Pollak et al. (2007), and Rennard et al. (2006) were not included in the Stead et al. analysis but were mentioned in the list of data sets excluded from their review. No reasons were given as to why these data sets were excluded. Lastly, the Dautzenberg (2007) and Kralikova (2009) papers replaced the abstracts utilized by Stead et al. Two of these 11 additional papers were subdivided into two data sets each, giving a total of 104 data sets derived from 87 different publications included in this analysis. All rejected studies are noted and referenced in the Appendix.

All of the data sets used for the meta-analyses are summarized in Table 1. Five different types of NRT were used, namely, gum (47 data sets), patch (36 data sets), sublingual tablets/lozenges (6 data sets), inhaler (6 data sets), and spray (4 data sets). In addition there were five data sets that used combinations of NRT. Twenty data sets were conducted in countries where the vast majority of cigarettes smoked utilize minimal added ingredients (flue-cured). Of these data sets, 14 were from the United Kingdom, 3 from Australia, 2 from New Zealand, and 1 from Canada. The remaining 84 data sets were from countries where cigarettes contain added ingredients (blended). A total of 54 data sets were from the United States, and the remaining 30 studies were from Europe. The European studies include seven from Sweden, six from Denmark, three from Croatia, two each from Belgium, Iceland, Italy, Spain, and Switzerland, and one each from the Czech Republic, Finland, France, and the Netherlands. The majority of the data sets were small, with 72 containing less than 300 subjects. There was no single study that made an unusually large contribution to the pooled results, with the largest study (ICRF, 1994, study 93), which had 842 treated subjects and 844 controls, contributing only 0.8% to the total sample. There are numerous other differences among the studies, and these will be discussed in greater detail in the next sections.

**Table 1:** List of data sets used for meta-analysis

Study No	Author and year	Total treated/controls	Quitters treated/controls	Type of NRT	Country	Level of support	Study quality	OR (95% CI)	Time Period	Def. of Abst.*	Comments
1	Ahluwalia et al. (2006)	189/189	29/28	Gum	US	High	A	1.04 (0.59–1.81)	6 months	PP	Motivational interviewing
2	Ahluwalia (2006)	189/188	55/45	Gum	US	High	A	1.22 (0.78–1.89)	6 months	PP	Health education
3	Cinciripini (1996)	32/32	12/7	Patch	US	High	B	1.71 (0.60–4.91)	1 year	PP	
4	Cooper (2005)	146/148	17/15	Gum	US	High	B	1.15 (0.55–2.39)	1 year	PP	
5	Fiore (1994)	44/43	15/9	Patch	US	High	B	1.63 (0.64–4.12)	6 months	PP	Patch for 8 weeks
6	Fiore (1994)	57/55	10/4	Patch	US	High	B	2.41 (0.71–8.15)	6 months	PP	Patch for 6 weeks
7	Fortmann (1995)	260/261	55/42	Gum	US	Low	B	1.32 (0.85–2.04)	12 months	PP	
8	Garvey (2000)	203/203	26/13	Gum	US	High	B	2.00 (1.00–4.00)	1 year	SA	4 mg gum
9	Garvey (2000)	202/203	27/13	Gum	US	High	B	2.08 (1.04–4.04)	1 year	SA	2 mg gum
10	Glover (2002)	120/121	22/12	Tablets	US	High	B	1.85 (0.88–3.90)	12 months	SA	
11	Gross (1995)	131/46	37/6	Gum	US	High	B	2.16 (0.86–5.46)	24 weeks	SA	
12	Hall (1985)	35/36	16/10	Gum	US	High	B	1.65 (0.66–4.12)	52 weeks	PP	
13	Hall (1987)	35/34	12/7	Gum	US	High	B	1.66 (0.59–4.73)	52 weeks	PP	
14	Hall (1987)	36/34	18/7	Gum	US	Low	B	2.43 (0.90–6.54)	52 weeks	PP	
15	Harackiewicz (1988)	90/47	12/9	Gum	US	Low	B	0.70 (0.27–1.77)	52 weeks	SA	
16	Hays (1999)	321/322	18/9	Patch	US	Low	B	2.01 (0.89–4.53)	24 weeks	SA	
17	Hughes (1989)	210/105	31/11	Gum	US	Low	A	1.41 (0.68–2.91)	1 year	SA	
18	Hughes (2003)	61/54	13/8	Patch	US	High	B	1.44 (0.55–3.73)	6 months	SA	
19	Hurt (1990)	31/31	9/8	Patch	US	High	B	1.12 (0.38–3.30)	56 weeks	SA	
20	Hurt (1994)	120/120	33/17	Patch	US	High	B	1.94 (1.03–3.67)	12 months	SA	
21	Jorenby (1999)	244/160	40/25	Patch	US	High	B	1.07 (0.62–1.83)	12 months	PP	
22	Joseph (1996)	294/290	41/32	Patch	US	High	A	1.26 (0.77–2.06)	24 weeks	PP	
23	Killen (1984)	22/20	5/6	Gum	US	High	B	0.76 (0.20–2.87)	10.5 months	PP	
24	Killen (1990)	301/309	57/56	Gum	US	High	B	1.04 (0.70–1.56)	12 months	PP	Ad lib gum
25	Killen (1990)	299/309	72/56	Gum	US	High	B	1.33 (0.90–1.95)	12 months	PP	Fixed gum
26	Killen (1997)	109/108	15/11	Patch	US	Low	B	1.35 (0.59–3.08)	12 months	PP	With video
27	Killen (1997)	103/104	21/14	Patch	US	Low	B	1.52 (0.73–3.14)	12 months	PP	Without video
28	Kinnunen (2008)	279/133	56/13	Gum	US	High	B	2.05 (1.08–3.89)	12 months	SA	Non-depressed
29	Kinnunen (2008)	126/70	19/4	Gum	US	High	B	2.64 (0.86–8.06)	12 months	SA	Depressed
30	Leischow (1996)	110/110	12/6	Inhaler	US	High	B	2.00 (0.72–5.52)	12 months	SA	
31	Lewis (1998)	62/62	6/4	Patch	US	Low	A	1.50 (0.40–5.58)	6 months	PP	
32	Mcgovern (1992)	146/127	50/39	Gum	US	High	C	1.12 (0.69–1.80)	12 months	PP	
33	Moolchan (2005)	34/40	6/2	Patch	US	High	A	3.53 (0.67–18.64)	6 months	PP	
34	Moolchan (2005)	46/40	4/2	Gum	US	High	A	1.74 (0.30–10.00)	6 months	PP	
35	Niaura (1999)	66/63	5/6	Gum	US	High	B	0.80 (0.23–2.74)	12 months	PP	
36	Okuyemi (2007)	66/107	5/10	Gum	US	High	B	0.81 (0.26–2.48)	6 months	PP	
37	Oncken (2007)	57/95	19/28	Patch	US	High	B	1.13 (0.58–2.21)	16 months	PP	
38	Oncken (2008)	100/94	11/9	Gum	US	High	C	1.15 (0.46–2.90)	6 months	PP	
39	Pirie (1992)	108/103	34/15	Gum	US	High	B	2.16 (1.11–4.20)	12 months	SA	Freedom from smoking program
40	Pirie (1992)	98/108	14/25	Gum	US	High	B	0.62 (0.30–1.25)	12 months	SA	Behavioral weight control program
41	Pollak (2007)	122/59	17/1	Various†	US	High	B	8.22 (1.07–63.27)	6 months	PP	
42	Rennard (2006)	215/214	17/5	Inhaler	US	Low	B	3.38 (1.23–9.34)	12 months	PP	
43	Sachs (1993)	113/107	28/10	Patch	US	High	B	2.65 (1.23–5.72)	12 months	SA	
44	Schneider (1983)	30/30	9/6	Gum	US	High	B	1.50 (0.48–4.74)	52 weeks	SA	Clinical support
45	Schneider (1983)	13/23	2/2	Gum	US	Low	B	1.77 (0.22–14.09)	52 weeks	SA	Low support
46	Schneider (1995)	128/127	23/10	Spray	US	High	B	2.28 (1.04–4.99)	1 year	SA	
47	Schneider (1996)	112/111	15/9	Inhaler	US	High	A	1.65 (0.69–3.93)	1 year	SA	
48	Shiffman (2009)	819/817	43/25	Gum	US	Low	A 1.72 (1.04–2.84)	6 months	SA	2 mg gum	
49	Shiffman (2009)	830/831	48/7	Gum	US	Low	A 6.86 (3.09–15.26)	6 months	SA	4 mg gum	
50	TNSG (1991)	249/253	65/31	Patch	US	High	B	2.13 (1.34–3.38)	24 weeks	SA	21 mg patch
51	TNSG (1991)	254/253	46/31	Patch	US	High	B	1.48 (0.91–2.41)	24 weeks	SA	14 mg patch
52	Westman (1993)	79/80	16/2	Patch	US	High	B	8.10 (1.80–36.40)	6 months	SA	
53	Wewers (2009)	147/155	13/0	Patch	US	High	B	Not calculable	12 months	PP	
54	Abelin et al. (1989)	100/99	18/12	Patch	Switzerland	Low	B	1.48 (0.68–3.24)	12 months	SA	6, 9, and 12 months results in Müller et al., 1990
55	Blondal et al. (1989)	92/90	30/22	Gum	Iceland	High	B	1.33 (0.72–2.48)	12 months	SA	
56	Blondal et al. (1997)	79/78	20/13	Spray	Iceland	High	A	1.52 (0.71–3.26)	1 year	SA	
57	Dautzenberg (2007)	211/222	25/18	Lozenge	France	Low	B	1.46 (0.78–2.76)	26 weeks	SA	
58	Dautzenberg (2007)	230/230	8/2	Lozenge	US	Low	B	4.00 (0.84–19.04)	26 weeks	SA	
59	Ehrsam (1991)	56/56	9/3	Patch	Switzerland	High	B	3.00 (0.77–11.67)	9 months	SA	
60	Fagerström (1982)	47/49	30/22	Gum	Sweden	High	B	1.42 (0.72–2.81)	6 months	SA	
61	Fagerström (1984)	50/22	14/3	Gum	Sweden	High	C	2.05 (0.54–7–87)	12 months	SA	High support
62	Fagerström (1984)	46/27	10/1	Gum	Sweden	Low	C	5.87 (0.71–48.41)	12 months	SA	Low support
63	Glavas et al. (2003a)	56/56	13/9	Patch	Croatia	Low	A	1.44 (0.57–3.65)	12 months	SA	
64	Glavas & Rumboldt (2003b)	40/40	15/6	Patch	Croatia	Low	A	2.50 (0.88–7.10)	6 months	SA	Patch for 3 weeks
65	Glavas & Rumboldt (2003b)	40/40	14/6	Patch	Croatia	Low	A	2.33 (0.82–6.68)	6 months	SA	Patch for 6 weeks
66	Hilberink et al. (2011)	243/148	18/5	Various‡	Netherlands	Low	B	2.19 (0.80–6.03)	12 months	PP	
67	Hjalmarson (1984)	106/100	31/16	Gum	Sweden	High	B	1.83 (0.94–3.54)	12 months	SA	
68	Hjalmarson et al. (1994)	125/123	34/18	Spray	Sweden	High	B	1.86 (1.00–3.47)	12 months	SA	
69	Hjalmarson et al. (1997)	123/124	35/22	Inhaler	Sweden	High	A	1.60 (0.89–2.89)	12 months	SA	
70	Jensen et al. (1991)	211/82	90/28	Gum	Denmark	High	B	1.40 (0.86–2.29)	6 months	SA	
71	Kornitzer et al. (1995)	149/75	27/10	Patch	Belgium	High	A	1.36 (0.62–2.96)	52 weeks	SA	Active patch and gum versus placebo gum
72	Kornitzer et al. (1995)	150/75	19/10	Patch	Belgium	High	A	0.95 (0.42–2.14)	52 weeks	SA	Active patch and placebo gum versus placebo gum
73	Kralikova et al. (2009)	209/105	39/9	Inhaler	Czech Rep.	High	B	2.18 (1.02–4.66)	12 months	SA	
74	Paoletti et al. (1996)	60/60	17/5	Patch	Italy	Low	B	3.40 (1.18–9.81)	52 weeks	SA	
75	Puska et al. (1995)	150/150	36/26	Patch	Finland	Low	B	1.38 (0.80–2.41)	52 weeks	SA	
76	Quilez Garcia et al. (1989)	37/38	13/5	Gum	Spain	High	B	2.67 (0.87–8.24)	6 months	SA	
77	Salvador Llivina et al. (1988)	113/103	54/28	Gum	Spain	High	B	1.76 (1.04–2.98)	12 months	SA	
78	Segnan et al. (1991)	294/275	22/15	Gum	Italy	High	A	1.37 (0.70–2.70)	1 year	SA	
79	Tonnesen et al. (1988)	60/53	23/12	Gum	Denmark	High	A	1.69 (0.77–3.73)	12 months	SA	
80	Tonnesen et al. (1991)	145/144	25/6	Patch	Denmark	High	A	4.14 (1.65–10.39)	12 months	SA	
81	Tonnesen et al. (1993)	145/141	22/7	Inhaler	Denmark	High	A	3.06 (1.27–7.38)	12 months	SA	
82	Tonnesen et al. (2006)	95/88	13/4	Tablet	Denmark	Low	B	3.01 (0.95–9.58)	12 months	SA	
83	Tonnesen et al. (2006)	90/97	13/6	Tablet	Denmark	High	B	2.34 (0.85–6.40)	12 months	SA	
84	Wallstrom et al. (2000)	123/124	28/19	Tablet	Sweden	High	A	1.49 (0.79–2.80)	12 months	SA	
85	Binnie et al (2007)	59/57	4/2	Various¶	UK	High§	C	1.93 (0.34–10.97)	12 months	PP	
86	BTS (1983)	400/402	40/56	Gum	UK	Low	B	0.72 (0.47–1.10)	12 months	PP	
87	Campbell et al. (1987)	424/412	13/9	Gum	UK	Low	B	1.40 (0.59–3.32)	12 months	SA	
88	Campbell et al. (1991)	107/105	21/21	Gum	UK	High	B	0.98 (0.51–1.90)	12 months	SA	
89	Campbell et al. (1996)	115/119	24/17	Patch	UK	High	B	1.56 (0.75–2.86)	12 months	SA	
90	Gilbert et al. (1989)	112/111	7/8	Gum	Canada	Low	B	0.87 (0.30–2.47)	12 months	SA	
91	Gourlay et al. (1995)	315/314	5/4	Patch	Australia	Low	B	1.25 (0.33–4.68)	6 months	SA	
92	Hand et al. (2002)	136/109	20/15	Various	UK	High	C	1.07 (0.52–2.18)	1 year	SA	
93	ICRF (1994)	842/844	76/53	Patch	UK	High	A	1.44 (1.00–2.07)	12 months	SA	
94	Jamrozik et al. (1984)	101/99	10/8	Gum	UK	Low	A	1.22 (0.46–3.23)	6 months	PP	
95	Jarvis et al. (1982)	58/58	18/8	Gum	UK	High	B	2.25 (0.91–5.58)	12 months	SA	
96	Malcolm et al. (1980)	73/63	17/3	Gum	UK	High	B	4.89 (1.37–17.46)	6 months	SA	
97	Molyneux et al. (2003)	91/91	10/4	Various#	UK	Low	B	2.50 (0.76–8.26)	12 months	SA	
98	Prapavessis et al. (2007)	26/27	4/4	Patch	New Zealand	High	B	1.04 (0.24–4.60)	52 weeks	SA	Cognitive therapy
99	Prapavessis et al. (2007)	33/35	9/3	Patch	New Zealand	High	B	3.18 (0.79–12.78)	52 weeks	SA	Exercise
100	Richmond et al. (1993)	200/150	17/14	Gum	Australia	High	C	0.91 (0.44–1.91)	6 months	SA	
101	Richmond et al. (1994)	156/157	39/19	Patch	Australia	High	A	2.07 (1.14–3.73)	6 months	SA	
102	Russell et al. (1983)	679/675	60/28	Gum	UK	Low	C	2.13 (1.34–3.38)	1 year	SA	
103	Stapleton et al. (1995)	800/400	77/19	Patch	UK	High	A	2.03 (1.21–3.40)	52 weeks	SA	
104	Sutherland et al. (1992)	116/111	30/11	Spray	UK	High	B	2.61 (1.25–5.46)	12 months	SA	

* PP, point prevalence; SA, sustained abstinence.

† Gum, patch, or tablet.

‡ NRT type cannot be specified, since subjects were responsible for choosing and purchasing their own treatment.

¶ Gum or patch.

§ High support for the treated group, low support for controls.

□Patch or inhaler.

# Gum, patch, inhaler, tablet, or spray.

For the logistic regression analysis of cessation rates, the analysis was conducted independently on the treatment groups and the control groups. The number of data sets used was slightly greater than the list shown in Table 1. The reason for this is that there were some treatment groups in the publications listed in Table 1 for which there was no separate corresponding control group, and some control groups for which there was no corresponding treatment group. Although these groups could not be included in the meta-analysis, they could be and were included in the logistic regression. In addition, it should be noted that, in some cases, a control group was used as the comparison for two treatment groups, in which case it was considered only once in the multiple logistic regression analysis. A total of five additional treatment data sets were included in the multiple logistic regression analysis, whereas one data set was not analyzed giving a final number of 108. These data sets are listed in Table 2 (A and B). A total of 10 additional control data sets were included in the multiple logistic regression analysis, whereas six data sets were not analyzed giving a final number of 108. These data sets are listed in Table 2 (C and D).

**Table 2:** List of additional or excluded treatment and control data sets

Study no.	Author and year	Number of participants	Number of quitters	Type of NRT	Country	Level of support	Study quality	Time period	Def. of Abst.*	Comments
*List of additional treatment data sets used for the multiple regression analysis*										
12a	Hall (1985)	42	15	Gum	US	Low	B	52 weeks	PP	Low support plus gum
16a	Hays (1999)	315	34	Patch	US	Low	B	24 weeks	SA	Open label with pay
76a	Quilez Garcia (1989)	31	8	Gum	Spain	High	B	6 months	SA	Follow-up individually with physician
79a	Tonnesen (1988)	60	16	Gum	Denmark	High	A	12 months	SA	Two additional treatment groups with 4 mg gum (27) and 2 mg gum (33)
100a	Richmond (1993)	100	12	Gum	Australia	Low	C	6 months	SA	GP advice plus nicotine gum
*List of treatment data sets excluded from the multiple regression analysis*										
74	Paoletti (1996)	60		Patch	Italy	Low	B	52 weeks	SA	Data not appropriate for multiple regression analysis
*List of additional control data sets used for the multiple regression analysis*										
15a	Harackiewicz (1988)	38	3		US	Low	B	52 weeks	SA	Short booklet distributed with tips to stop smoking
31a	Lewis (1998)	61	3		US	Low	A	6 months	PP	Minimal counseling
78a	Segnan (1991)	62	3		Italy	Low	A	1 year	SA	Minimal intervention
78b	Segnan (1991)	292	19		Italy	High	A	1 year	SA	Repeated counseling with spirometry
86a	BTS (1983)	377	41(2)		UK	Low	B	12 months	SA	Verbal advice from physician, stop smoking booklet
86b	BTS (1983)	371	41(3)		UK	Low	B	12 months	SA	Verbal advice from physician
87a	Campbell et al (1987)	149	2		UK	Low	B	12 months	SA	Additional control group
96a	Malcolm (1980)	74	10		UK	High	B	6 months	SA	Additional control group
97a	Molyneux (2003)	92	7		UK	Low	B	12 months	SA	Usual care
102a	Russell (1983)	584	23		UK	Low	C	1 year	SA	No contact with physician
*List of control data sets excluded from the multiple regression analysis*										
9	Garvey (2000)	203	13		US	High	B	1 year	SA	Same control group as used for data set 8
34	Moolchan (2005)	40	2		US	High	A	6 months	PP	Same control group as used for data set 33
51	TSNG (1991)	253	31		US	High	B	24 weeks	SA	Same control group as used for data set 50
72	Kornitzer (1995)	75	10		Belgium	High	A	52 weeks	SA	Same control group as used for data set 71
74	Paoletti (1996)	60	5		Italy	Low	B	52 weeks	SA	Data not appropriate for multiple regression analysis
75	Puska (1995)	0	0		Finland	Low	B	52 weeks	SA	Both the treatment group (patch) and the control group were encouraged to use gum. Therefore, the entire sample was considered as a treatment group.

* PP, point prevalence; SA, sustained abstinence.

### Effect of NRT

Although not the primary focus of the present study, the results of this analysis should clearly demonstrate a positive effect of NRT intervention to confirm that the selection of the data sets shown in Table 1 still correspond to the data sets used by Stead, et al. This is indeed the case. When the total data set was analyzed, the pooled OR comparing smoking cessation rates for treated subjects and for control subjects was 1.70 (95% CI, 1.58–1.82) by fixed effects meta-analysis and 1.76 (95% CI, 1.61–1.93) by random effects meta-analysis (Table 3). As there is heterogeneity in this pooled OR, as demonstrated by the I^2^ value (*p* = 0.006 on 103 degrees of freedom) (Higgins et al., 2003), and in many of the ORs estimated in the stratified analyses as well, conclusions drawn from these meta-analyses will be based on the random-effects estimates, although pooled estimates using both fixed and random effects methods for all comparisons are listed in Table 3. The pooled ORs for NRT intervention are somewhat higher than the estimate of 1.58 (95% CI, 1.50–1.66) reported by Stead et al., from which many of the data sets used in this analysis were drawn. The Stead et al. estimate, however, was based on pooled risk ratios using Mantel-Haenzel methodology. Using the exact same methodology, the pooled fixed effects estimate for NRT intervention of the 104 data sets used here was 1.58 (95% CI, 1.49–1.68), which is identical to that reported in the review. This result clearly indicates that NRT does indeed improve individuals' ability to quit smoking, at least under the conditions of a clinical trial, and also clearly demonstrates that the data set used to address the comparisons made in this analysis is representative.

**Table 3:** Estimates of treatment effects on cessation rates, overall as well as stratified by type of NRT, type of cigarette, study duration, type of abstinence, level of support, study decade, study size, study quality, as well as combined strata, regarding type of cigarette and type of abstinence

Description	No. of studies	Fixed effects pooled OR (95% CI)	Random effects pooled OR (95% CI)	I^2^	Degrees of freedom	*p*
All studies	104	1.70 (1.58–1.82)	1.76 (1.61–1.93)	0.28	103.00	0.006
Nicotine gum	47	1.52 (1.38–1.69)	1.61 (1.39–1.86)	0.44	46.00	0.001
Nicotine patch	36	1.80 (1.59–2.04)	1.81 (1.59–2.06)	0.05	35.00	0.383
Nicotine tablets/lozenge	6	1.95 (1.39–2.74)	1.95 (1.39–2.74)	0.00	5.00	0.722
Nicotine inhaler	6	2.30 (1.65–3.21)	2.30 (1.65–3.21)	0.00	5.00	0.784
Nicotine spray	4	2.34 (1.62–3.34)	2.34 (1.62–3.34)	0.00	3.00	0.789
Blended countries	84	1.75 (1.61–1.90)	1.80 (1.63–1.98)	0.20	83.00	0.066
Flue-cured countries	20	1.53 (1.31–1.79)	1.61 (1.27–2.04)	0.50	19.00	0.007
1-year cessation data	73	1.66 (1.52–1.80)	1.71 (1.54–1.90)	0.24	72.00	0.037
6-month cessation data	31	1.81 (1.58–2.08)	1.91 (1.59–2.30)	0.36	30.00	0.025
Sustained abstinence	72	1.94 (1.78–2.12)	1.96 (1.78–2.16)	0.14	71.00	0.16
Point prevalence	32	1.30 (1.15–1.48)	1.32 (1.15–1.51)	0.09	31.00	0.322
High support	73	1.69 (1.55–1.84)	1.73 (1.57–1.91)	0.22	72.00	0.051
Low support	30	1.72 (1.49–1.98)	1.88 (1.53–2.30)	0.42	29.00	0.009
Studies, 1980–1989	24	1.70 (1.44–2.02)	1.82 (1.45–2.28)	0.37	23.00	0.037
Studies, 1990–1999	46	1.64 (1.48–1.80)	1.68 (1.49–1.90)	0.30	45.00	0.029
Studies, 2000–2010	34	1.83 (1.59–2.12)	1.88 (1.60–2.22)	0.18	33.00	0.183
>300 subjects	32	1.57 (1.42–1.74)	1.64 (1.42–1.89)	0.47	31.00	0.002
<300 subjects	72	1.84 (1.66–2.05)	1.86 (1.67–2.08)	0.11	71.00	0.227
Quality A	26	1.73 (1.52–1.98)	1.79 (1.52–2.11)	0.28	25.00	0.091
Quality B	70	1.71 (1.56–1.88)	1.80 (1.60–2.02)	0.31	69.00	0.009
Quality C	8	1.51 (1.18–1.94)	1.51 (1.17–1.95)	0.03	7.00	0.403
Blended and SA	55	2.03 (1.82–2.25)	2.04 (1.82–2.27)	0.08	54.00	0.315
Blended and PP	29	1.38 (1.21–1.57)	1.38 (1.21–1.57)	0.00	28.00	0.647
Flue cured and SA	17	1.74 (1.47–2.06)	1.75 (1.42–2.16)	0.27	16.00	0.145
Flue cured and PP	3	0.79 (0.54–1.17)	0.85 (0.52–1.38)	0.15	2.00	0.308

An important comparison, particularly from the standpoint of this analysis, is the effect of NRT intervention between countries in which blended cigarettes (contains added ingredients) are primarily smoked compared to countries in which flue-cured cigarettes (no or few added ingredients) are primarily smoked. For the 84 studies reported from the United States and Europe excluding the United Kingdom (blended cigarettes), the pooled random effects OR estimate was 1.80 (95% CI, 1.63–1.98), while the pooled random effects OR estimate forflue-cured countries was 1.61 (95% CI, 1.27–2.04). The estimate for blended cigarettes did not indicate heterogeneity, with *p* = 0.066 on 83 degrees of freedom. However, the results for flue-cured countries remained heterogeneous, with *p* = 0.007 on 19 degrees of freedom. This comparison suggests that NRT intervention in smokers of cigarettes containing ingredients may be about 12% more efficacious than in smokers of cigarettes containing minimal ingredients, although this difference is clearly not statistically significant (*p* = 0.39).

Table 3 contains the results obtained from stratification of intervention effects estimates, according to other variables that were extracted from the publications. Always compared to the respective reference category, the results indicate larger NRT effects in data sets in which results were reported after 6 months as opposed to 1 year, when sustained abstinence is considered (both overall as well as when types of cigarettes were considered separately), when support was low, when the study was conducted b efore or after 1990–1999, and when study quality was A or B. With respect to the efficacy of the different types of NRT, the lowest pooled OR is for nicotine gum, with increasing effectiveness being associated with the nicotine patch, nicotine tablet/lozenge, nicotine inhaler, and nicotine spray. Only the difference between nicotine spray and nicotine gum is statistically significant (*p* = 0.03).

### Cessation rates in blended versus flue-cured countries

Model selection based on both forward and backward selection through stepwise logistic regression, carried out in control group data, resulted in study duration and study quality not being retained in the final main effects model. The final main effects model contained the variables cigarette type (blended versus flue-cured as reference), cessation type (sustained abstinence versus point prevalence as reference), support (high versus low psychological support as reference), decade (ordinal with levels 0, 1, and 2 representing the periods 1980–1989, 1990–1999, and 2000–2010, respectively), and study size (≥150 versus <150 as reference). Screening for bivariate interactions among the variables contained in the final main effects model resulted, in both forward and backward selection, in the following four additional interaction terms being retained in the final model: cigarette type versus support, cigarette type versus decade, study size versus decade, and study size versus cessation type. To account for the effect of study decade on cessation rates not being linear, the ordinal variable was replaced by a pair of dummy variables with period 1980–1989 as reference. Table 4 contains the effect estimates of the main effects model, as well as of the interaction model for the control group data. To facilitate assessing the stability of the estimates based on the main effects model, they were also calculated for the treatment group data.

**Table 4:** Effect estimates and 95% CI based on the main effects model in control and treatment group data. Effect estimates of the interaction model are given for the control group data on the right half, indicating the scope of applications of estimates (population restrictions), according to the interaction structure of the model. In the effect column, the exposure category is mentioned

Main effects model			Interaction model			
	
Effect	OR	95% CI	Effect	Population restrictions	OR	95% CI
**Data: Control groups**			**Data: Control groups**			
Blended cigarettes	1.32	1.14–1.53	Blended cigarettes	Low support and 1980–1989	3.47	2.20–5.45
Sustained abstinence	0.49	0.44–0.55	Blended cigarettes	Low support and 1990–1999	1.52	0.95–2.42
High support	2.38	2.06–2.75	Blended cigarettes	Low support and 2000–2010	1.05	0.39–2.82
1990–1999	0.58	0.50–0.69	Blended cigarettes	High support and 1980–1989	2.12	1.41–3.20
2000–2010	0.42	0.35–0.50	Large study	Point prevalence and 1980–1989	2.19	1.39–3.44
Large study	0.71	0.63–0.80	Large study	Point prevalence and 1990–1999	1.04	0.84–1.30
**Data: Treatment groups**			Large study	Point prevalence and 2000–2010	1.26	0.96–1.65
Blended cigarettes	1.90	1.70–2.13	Large study	Sustained abstinence and 1980–1989	0.97	0.62–1.52
Sustained abstinence	0.89	0.81–0.98	High support	Flue-cured cigarettes	3.22	2.12–4.88
High support	2.02	1.82–2.25	1990–1999	Flue-cured cigarettes and small study	1.18	0.77–1.80
1990–1999	0.62	0.55–0.70	2000–2010	Flue-cured cigarettes and small study	1.16	0.71–1.87
2000–2010	0.45	0.39–0.51	Sustained abstinence	Small study	0.70	0.58–0.83
Large study	0.73	0.66–0.80				

As the effect estimates of the main effects model using control group data demonstrate, cessation rates were about 30% higher in countries where predominantly blended cigarettes (ingredients) were smoked, as compared to countries where predominantly flue-cured cigarettes (limited ingredients) were smoked. Cessation rates were lower by a factor of two when determined through sustained abstinence, as compared to point prevalence. High levels of psychological support were associated with an almost 140% increase of the chances of successful cessation, compared to low support levels. Compared to the 1980–1989 period, chances of successful cessation were reduced by about 70% compared to the 1990–1999 decade and by more than a factor of two compared to the most recent decade. In larger data sets, the probability of cessation was about 40% lower than in smaller data sets.

Overall, this pattern of effects of the variables of the main effects model was replicated in the treatment group data set. While, here, the effect related to cigarette type was more pronounced (OR 1.90 for treatment group compared to OR 1.32 in the control group), the cessation type (sustained versus point prevalence cessation) as well as the level of psychological support was of somewhat less importance. The estimates related to study period and study size were essentially equivalent to those obtained in the control group data.

The interaction structure identified by screening for bivariate interactions poses considerable restrictions on what effects can be estimated in a meaningful way, which implies a rather complicated pattern of effect estimates. It needs to be noted that assessing the estimates obtained for the interaction model is conditional to the restrictions as contained in Table 4, since estimates are either conditional to reference levels or may imply effect modification by other variables, or a combination of both. In low psychological support data sets, cessation rates were higher by almost 250% in countries where predominantly blended cigarettes as compared to flue-cured cigarettes were smoked, and in the decade of 1980–1989, whereas (in the same decade) the excess was less than half that size under conditions of high psychological support. The profound excess cessation rates in the 1980–1989 decade in blended countries, observed in data sets with low psychological support, declined through decades 1990–1999 and 2000–2010, with no substantial difference left for the most recent decade. Restricted to data sets in countries in which predominantly flue-cured cigarettes are smoked, high levels of psychological support were associated with increased cessation rates by about 220%, compared to low support levels. Decade *per se* did not seem to have an impact on cessation rates in flue-cured countries based on results from small studies.

Restricted to point prevalence data, large study size did not have an impact on cessation rates in the 1990–1999 and 2000–2010 decades, compared to the 1980–1989 decade, where cessation rates in large data sets were higher by almost 120% compared to small studies. No effect of study size on cessation rates was observed in the 1980–1989 decade when the outcome was sustained abstinence. When assessed in small data sets, sustained cessation rates were lower by about 30% as compared to point prevalence rates.

A combined analysis of control and treatment groups data was undertaken by means of conditional logistic regression based on the main effects model, with study number as strata and a variable indicating type of group (treatment versus control). The obtained treatment effect, that is, adjusted for all variables of the main effects model as contained in Table 4 was 1.77 (95% CI: 1.60–1.96), indicating a 77% increased cessation odds under nicotine replacement treatment, as compared to control group conditions. The estimates obtained for the variables of the main effects model were very close to those contained in Table 4: 1.85 (1.45–2.37) for blended cigarettes, 0.79 (0.63–0.99) for sustained abstinence, 2.11 (1.64–2.73) for high support, 0.56 (0.44–0.71) for period 1990–1999, 0.44 (0.33–0.57) for period 2000–2010, and 0.84 (0.67–1.04) for large study size.

Lastly, to explore the effects of different types of nicotine replacement therapies, the analysis of the treatment group data set was repeated by adding a set of reference-coded dummy variables to the main effects model, which coded different types of nicotine replacement against nicotine spray as reference. The estimates of the main effects model variables changed by <3%, except for the 1990–1999 period effect, which was 0.66 (data not shown) instead of 0.62, as shown in Table 4. The OR were 0.93, 0.82, 0.95, and 0.87 for gum, patch, lozenge, and inhaler, respectively, indicating somewhat lower effects for all four NRTs compared to nicotine spray, but with 95% confidence intervals including unity.

**Figure 1:**
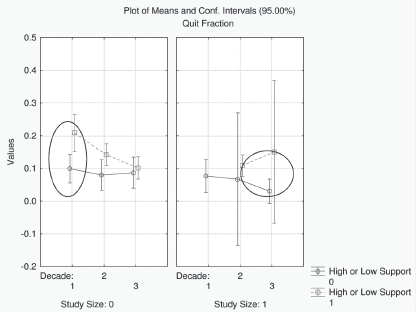
In small studies (left panel), studies with high support resulted in increased levels of smoking cessation as compared to studies with low support in decade 1. The difference in smoking cessation rates as a function of support declined in the 1990s (decade 2) and 2000s (decade 3). In large studies (right panel), there was a significant effect of high support in studies conducted in the 2000s but not in studies conducted in the 1990s. (See colour version of this figure online at http://www.informahealthcare.com/iht)

Because of the complexity of the interaction structure that was obtained when using stepwise logistic regression, it was decided to also analyze the data using a CTA approach to see if a simpler picture emerged. Only two factors were found to be differentiated when this analysis was applied to the control group, namely, the study size, and the level of support. The results for level of support was found to be significantly differentiated in the CTA, with quitting being higher in data sets where subjects received a high level of psychological support (14%) than in those where subjects received a low level of support (8%), which was in line with the multiple logistic regression analysis. Also consistent with the main effects logistic regression model was the fact that the quit fraction was statistically higher in small data sets (>150, 13%) than in large data sets (<150, 9%). No difference in cessation rates was detected between smokers in blended countries (ingredients) compared to smokers in flue-cured countries (few or no ingredients). In addition, there was no difference in quit rates with respect to whether results were reported by sustained abstinence or point prevalence. Both of these results are in contrast with the multiple logistic regression results. Although there was no effect of decade, there was an interaction between decade, study size and level of support. Figure 1 shows a plot of the relationship between data sets with high support and those with low support plotted as a function of decade for both small data sets and large data sets. As shown in the figure, the difference in cessation rates between small data sets with high and low support was statistically different only between 1980 and 1989. The difference narrowed considerably in the following decade, and in the 2000–2010 decade, there was essentially no difference as a function of level of support.

## Discussion

### Quitting rates in countries with added ingredients (blended) and those with few added ingredients (flue-cured)

The main analysis reported here has looked at 108 data sets derived from similar control groups from clinical trials on NRT performed over the last three decades in seven different countries. If the use of ingredients would have increased the addictiveness of cigarettes, a lower success rate for cessation in these trials would have been expected in the countries where the addition of ingredients to cigarettes predominates (i.e., in blended markets). This was clearly not the case for the analysis reported here. The results of the main effect model obtained from multiple logistic regression analysis of the 108 data sets indicate that cessation rates were generally higher in countries with cigarette ingredients (blended) than those with limited or no ingredients (flue-cured). Although this finding seems to be fairly robust, in that it is also reflected in a similar group of data sets derived from the treatment groups, and remains significant through a number of interaction models as well, it would seem implausible to suggest that it is the presence of ingredients that make it easier to quit. It is, however, clear that the data reported here do not in anyway support the suggestion that ingredients could increase the addictiveness of cigarettes.

A number of variables were identified as influencing cessation rates as shown in Table 4. Trials with high psychological/behavioral support were significantly more successful in leading to smoking cessation than were trials with low support. This difference is larger in the control group than in the NRT group, possibly because the individuals in the intervention group benefited from both NRT and the presence of psychological or behavioral support, although the difference did not reach statistical significance at the 5% level (*p* = 0.09). Small data sets reported a greater extent of smoking cessation than did large data sets, and this result was statistically significant for both the treatment and the control groups in the main effects model. This difference may well reflect an effect of more focused psychological support: individuals in small data sets are more likely to receive individual attention than individuals in large data sets. The results for the method of reporting cessation, sustained abstinence, or point prevalence, are completely in line with expectations, in that reported cessation rates were lower when sustained abstinence was reported than when point prevalence was used. It is important to note, as it is detailed in Table 4, that the adjusted OR for sustained abstinence with respect to point prevalence in the NRT group was 0.89 (95% CI, 0.81–0.98), whereas for the control group the OR was 0.49 (95% CI, 0.44–0.55). This difference is statistically significant (*p* = 3.3 × 10^−15^). Why such a difference emerged between the NRT group and the control group with respect to reporting method is certainly not obvious.

Considering that all the trials were conducted between 1980 and 2010, it seemed logical to divide the data sets into three time periods, namely 1980–1989 (24 data sets), 1990–1999 (46 data sets), and 2000–2010 (34 data sets). Therefore, decade is a ternary variable, while all the other variables are binary. For both the treatment and control group cessation rates declined as a function of decade. The result shown in Table 4 can be interpreted as a decrease by about a factor of 2.4 in cessation rates when moving from the 1980s through the 1990s, and then to the first 11 years of the 21st century. This finding would appear to be counter-intuitive. During this 30-year period, there has been an increase in both external pressures to quit smoking and in public smoking bans in all countries included in this analysis. As a consequence, it might be anticipated that cessation rates should have increased during this period of time, yet the opposite is true, at least in the setting of a randomized clinical trial to evaluate NRT. On the other hand, this result appears to be in line with population-based survey data suggesting that individuals who continue to smoke are far more refractory than those who have already quit. A quotation from NCI Smoking and Tobacco Control Monograph 15 (US Department of Health and Human Services, 2003) clearly supports this.

The fraction of those who have ever smoked but have successfully quit increased dramatically over the last half-century to the point at which approximately one-half of those who have ever smoked are currently former smokers. However, declines in per capita consumption slowed dramatically during the middle of the 1990s, and the CPS data show a decline in cessation attempts and abstinence between the 1992/93 and 1995/96 surveys (see Chapter 8). These observations raise a concern that those smokers who could easily quit, or who could be influenced by existing tobacco control approaches to quit, have done so, leaving behind a residual population of smokers who are more heavily addicted and who need new or more individualized cessation interventions.

A number of statistically significant interactions were identified in the stepwise logistic regression analysis, two of which involved decade (Table 4). Of particular note is the observation that the higher quit rates for smokers of blended cigarettes declined during the three decades, so that in the 1990–1999 and 2000–2010 intervals, there was no longer a statistically significant difference between quit rates for smokers of blended cigarettes compared to smokers of flue-cured cigarettes; however, this was observed only for data sets characterized by low psychological/behavioral support. A possible explanation is that this result is simply a consequence of the restrictions imposed by the interaction model resulting in a higher influence of random variation in the comparisons. This is supported by the fact that there are several other interaction terms involving decade. For example, although the main effects model demonstrates that cessation rates for large data sets are statistically lower than those in small data sets, cessation rates for large data sets using point prevalence as the reporting method were significantly higher in the decade 1980–1989. This effect was also seen in the latter two decades, although in this case the difference was not statistically significant. Likewise, the decrease in cessation rate as a function of decade observed in the main effects model is not observed in the interaction of decade, flue-cured cigarettes, and small data sets.

Not only did the interaction screening produce a very complex picture but it was also observed that small corrections to the data set resulted in noticeable changes in the interaction structure. As a consequence, the control group data were also investigated using a CTA. The advantage of CTA is that a much simpler picture is produced. In that the data are split in a recursive fashion, each split determines what can be split in the subsequent stages. Since this process is a one-variable-at-a-time analysis, combinations of variables are not considered during the splitting but rather emerge based on the tree structure. This implies that when a certain split is undertaken – due to the superior effect size of a particular variable at any particular stage – the possibilities to detect interactions further down are very much limited. This is also a weakness of CTA, since effects that might be detected by a technique, such as multiple logistic regression analysis, may not emerge as being significant. This issue as well as others is discussed in detail in a recent review (Strobl et al., 2009). Indeed, a much simpler picture with respect to interactions was observed by CTA with only one statistically significant interaction, a three-way interaction, being observed. Cessation rates in small data sets with high support declined as a function of decade when compared to small data sets with low support, with no statistically significant difference being observed in the most recent decade (Figure 1). This is exactly the same pattern that was observed using multiple regression analysis with respect to cessation rates of blended and flue-cured smokers with low support as a function of decade. This CTA interaction result is most probably a consequence of random differences with respect to interactions with decade, since there is no logical reason that can explain why level of support ceased to become important as a function of decade.

Perhaps not unsurprisingly, given the properties of CTA noted above, only two variables that had a statistically significant effect on cessation rates were identified by this analysis. Cessation rates in studies with a high level of psychological support were 80% higher than those with a low level of psychological support. This result is in agreement with the multiple logistic regression analysis, although the magnitude is somewhat less (Table 4). Also consistent with the logistic regression analysis, CTA showed that cessation rates in small data sets were significantly higher than those in large data sets. All other factors identified as having an effect on cessation rates in the stepwise regression were not differentiated in the CTA, including the difference between cessation rates for smokers of blended and flue-cured cigarettes. The failure to differentiate cessation rates as a function of type of cigarette smoked is not surprising, given the lower sensitivity of the CTA approach, particularly given that the difference observed by multiple logistic regression analysis, albeit statistically significant, was certainly not large (30%). Nevertheless, the finding by CTA clearly does not support a conclusion that cessation rates for smokers of cigarettes containing few or no ingredients (flue-cured) was increased compared to smokers of cigarettes containing multiple ingredients (blended).

### Effect of NRT

The meta-analysis conducted on the 104 data sets listed in Table 1 clearly indicates that the use of NRT can increase cessation rates. Moreover, there is complete agreement with the results of this analysis and the analysis carried out by Stead et al., as noted in the “Results” section. Although this analysis found a significant difference between nicotine spray and nicotine gum, the results of the logistic regression analysis indicate no statistical difference among any of the interventions. This is consistent with the findings of Stead et al., who concluded that, “The choice of which form [of NRT] to use should reflect patient needs, tolerability, and cost considerations.”

All of the variables that were evaluated as potential confounders in the multiple logistic regression analysis were also tested by meta-analysis (Table 3). The level of psychological support, which had been shown to be a statistically significant predictor of smoking cessation rates for both the control and treatment groups by multiple logistic regression (Table 4), was not statistically differentiated in the meta-analysis (*p* = 0.47), a finding in agreement with the conclusions of Stead et al. This lack of difference as a function of level of psychological support demonstrates that a subgroup analysis within a meta-analysis designed to measure the effect of one intervention on cessation rates, in this case NRT, does not predict cessation rates with respect to differences for other factors. As calculated by the multiple logistic regression analysis, cessation rates increase by a factor of about two when data sets with high support are compared to those with low support. However, because this increase in cessation rates occurs to about the same extent for both treatment and control groups, no statistically significant difference is observed in the meta-analysis.

A second interesting meta-analytical result involved the type of abstinence reported. A total of 72 data sets reported sustained abstinence; that is, continuous abstinence since the date that the individual reported having stopped smoking. The remaining 32 studies reported cessation by point prevalence. This type of evaluation utilized a fixed period of time, usually 1 week, during which a subject was abstinent just before being evaluated. As a consequence, lapses during which the subject may have been smoking were ignored. Therefore, itwouldbe anticipated that a greater success of NRT intervention would appear to have been obtained when cessation was determined by point prevalence, which was clearly demonstrated to be the case by logistic regression (Table 4). However, the pooled random effects OR for sustained abstinence of 1.96 (95% CI, 1.78–2.16) is clearly significantly larger than the pooled random effects OR of 1.32 (95% CI, 1.15–1.51) for point prevalence (*p* = 3.5 × 10^−6^). This difference was not observed by Stead et al., although they assessed the effect of type of abstinence using a different approach. The explanation for this apparently counter-intuitive finding can be seen from the results of the multiple regression analysis, which clearly show that there is a significant difference between the control group and the treatment group when comparing sustained abstinence to point prevalence, with the control group showing much lower cessation rates when assessed by sustained abstinence. This translates into a higher estimate for sustained abstinence than that for point prevalence by meta-analysis. This example once again demonstrates that the results of a meta-analysis cannot be used to assess the effect of a confounding factor on cessation rates.

A final point regarding this comparison is that the heterogeneity observed in the meta-analytic estimates can be explained by type of abstinence reported, since I^2^ values for both of these factors were not significant (sustained abstinence, *p* = 0.16 on 71 degrees of freedom; point prevalence, *p* = 0.29 on 31 degrees of freedom). As a consequence, it was of interest to determine the pooled ORs for smokers of blended cigarettes and flue-cured cigarettes, which was not differentiated in the meta-analysis, as a function of this variable. As noted above, although no heterogeneity had been observed in the pooled OR for blended data sets, the pooled OR for flue-cured data sets was heterogeneous. When this calculation was carried out, no significant heterogeneity was observed in any of the pooled ORs with *p* = 0.32 on 54 degrees of freedom for blended and sustained abstinence; *p* = 0.65 on 28 degrees of freedom for blended and point prevalence; p = 0.15 on 16 degrees of freedom for flue-cured and sustained abstinence; and *p* = 0.31 on 2 degrees of freedom for flue-cured and point prevalence. The relevant ORs are listed in Table 3, and the close agreement between the fixed effects estimate and the random effects estimate confirms the lack of heterogeneity. As with the complete analysis of the effect of NRT on smoking cessation for smokers of blended and flue-cured cigarettes, the pooled ORs for blended smokers remained higher than that for flue-cured smokers in both subgroups, although the differences were not statistically significant.

As indicated, although the pooled OR for smokers of blended cigarettes was greater (1.80) than that for smokers of flue-cured cigarettes (1.61), the difference was not statistically significant (*p* = 0.39). There were also no statistical differences for all other potential confounding factors that were evaluated in the logistic regression analysis, including the decade during which a study was conducted, study size, the time period at which abstinence was checked (6 months or 1 year), and study quality.

## Conclusions

The main conclusion resulting from this analysis is that there is no evidence that cessation rates for smokers of blended cigarettes, which contain a numbers of ingredients, including a mixture of flavorants, are any lower than those for smokers of flue-cured cigarettes, which contain few ingredients and no flavorants. Despite the fact that multiple logistic regression analysis did indeed determine that there was a small but statistically significant difference in cessation rates that favored smokers of blended cigarettes, it would be difficult to consider this result as being a real effect. At this time, there is no rational explanation that would suggest that addition of a number of ingredients to cigarettes would increase the ease of smoking cessation. However, all the data are consistent with the conclusion that the presence of ingredients currently being added to tobacco does not increase inherent cigarette addictiveness.

The major strength of this study is that three different approaches were utilized to analyze the data, providing considerable confidence in the final conclusions. Second, the data sets analyzed allow comparison of at least some of the results of this study with already published results, thereby also supporting the conclusions reached in this analysis. However, the choice of these data sets is also a weakness of this study. Due to the restriction of the data sets to those investigating only the use of NRT, as well as the fact that the control group had to be matched to the treatment group with the exception of NRT use, there were undoubtedly a number of data sets that could have been included in the multiple logistic regression analysis that were not included. On the other hand, there were 108 control data sets included in the multiple logistic regression analysis with more than 100,000 subjects. It is highly unlikely that inclusion of additional data sets would have changed the conclusions reached by this analysis. Another possible limitation of this study is that there were a number of other factors that could have contributed to differences in cessation rates; for example, methods of recruitment and study setting that could not be evaluated. It should be noted that there could be cultural differences that could influence smoking cessation rates between countries in which cigarettes without ingredients are smoked compared to those in which cigarettes with ingredients are smoked. Every attempt was made to minimize such cultural differences in that the studies that were used were all drawn from developed countries with generally similar cultural backgrounds.

This analysis also confirms a number of other factors of importance with respect to smoking cessation that have been previously reported. The results clearly demonstrate that NRT is effective, at least in the setting of a clinical trial, in increasing the probability of quitting smoking. The pooled ORs for all types of NRT is in exact agreement with the Stead et al. review when the same meta-analytic methodology is used, even though there was not a complete overlap of data sets used in the two analyses. Second, this analysis demonstrates that a high level of psychological support provides a clearly significant benefit with respect to smoking cessation above and beyond the effect of NRT. The role that various types of psychological or behavioral support can play with respect to smoking cessation has been known for many years (Schwartz, 1979), and there have been a number of recent meta-analyses that clearly demonstrate this (see Schmelzle et al., 2008, for a summary). However, this analysis is the first to demonstrate the importance of psychological/behavioral support using multiple logistic regression analysis. Lastly, the method used to determine smoking cessation, sustained abstinence, or point prevalence, provides results that are clearly statistically distinguishable, and, moreover, this factor is responsible for the heterogeneity observed in the various pooled ORs. This finding is consistent with the manner in which these reporting methods are defined, and data sets included in this analysis clearly demonstrate that when results using both methods are presented, the cessation rates are invariably higher for reporting by point prevalence than by sustained abstinence. However, no multistudy analysis has been previously published. Both this analysis and the Stead et al., review used sustained abstinence as the default evaluation, and it might be suggested that any future studies evaluating smoking cessation use sustained abstinence as the reporting method. It is interesting to note that almost all European studies and studies in flue-cured countries used sustained abstinence to report results, whereas only about 50% of American studies used sustained abstinence.
